# Editorial Chit-Chat

**Published:** 1875-10

**Authors:** 


### Editorial Chit-Chat.

W. F. C. may have written somewhat scathingly of that "art gallery" but many think him right.

PHOTOGRAPHY.—The very best out-door views we have ever seen taken are made by Van Aken, of this city.

NEW "LOCAL."—Mr. Will H. Perry is the spicy paragraphist of the *Gazette;* our evening paper sparkles with his wit.

PARK CHURCH.—This immense edifice, of which Rev. Thomas K. Beecher is pastor, will be regularly opened for religious worship this month. The building is one of the largest, finest, costliest and queerest in the state.

THE COLORED COMPANY.—It is thought that a prominent Banker—a general, will be tendered the command of the colored troops, (a company of which is being equipped in this city, by the bankers,) provided they "fight bravely," as usual.

MAILING MACHINE.—The present number of the BISTOURY is mailed by means of a new mailing machine, which promises to do its work speedily and well. How it performs, we will be able better to tell in our next.

ENTERTAINING.—If you desire to be profitably entertained and instructed, a splendid opportunity is offered in listening to Mr. Beecher's capital lectures upon the history of the Bible, at Park Church lecture room every Friday night.

BISTOURY WANTED.—We desire a few numbers of the BISTOURY for April, 1871. Any of our readers who have this number, and who do not desire to retain it for binding purposes, will be credited 25 cents upon subscription, by forwarding it to us.

THE STATE FAIR.—By the time this number of our Journal reaches its readers, our State Fair will be in full blast, with its big pumpkins, squashes, and other vegetables, and its endless machines and contrivances to benefit and extort money from the people. Some of this apparatus will be useful, much indifferent, and more useless. Yet, we expect to take our country cousins and all go to the big show.

SIXTEEN IN ONE BED.—A report to the Board of Health in this city, announces that there is a house of two rooms, in the Third Ward, inhabited by sixteen individuals, and but one bed in the house. Fritz wonders how those sleeping on the *outside* keep from rolling off.

VICK.—James Vick, the florist, of Rochester, publishes a magnificent quarterly magazine, named "Vicks Floral Guide," which is one of the finest illustrated and printed journals that comes to our table. Every lover of flowers should send for a copy and learn how to prepare for a splendid display of flowers next summer.

THE LECTURE COURSE.—We are glad to learn that the annual lecture course has been arranged for this winter and that an entire new array of tallent has been secured. This is as it should be. We believe the people have grown tired of the old party who have been accustomed to visit us nearly every every winter, for years. A new deal will be appreciated and enjoyed.

ASKED FOR A LETTER.—A chap called at the post office to-day, poked his head through one of those holes in the sash, and seeing George Whiton, enquired, "Say, mister, got any letters for Mike Howe?" "No," says George, "have'nt got any letters for *your* cow or any other fellow's cow,—get out!" And Mike left, wondering why George should accuse him of enquiring for a letter for his cow. At last account, he had not yet sovled the problem.

ELMIRA DRIVING PARK.—Elmira does not mean to be surpassed by any city in the facilities that go to make up a first-class and enterprising place for residence. Side by side with Eldridge Park and the State Fair grounds we now have the Driving Park, inaugurated and controlled by our wealthiest and most prominent citizens. The track is said to be the best in this state, while the grand stand, judge's stand, stables and hotel are unsurpassed in arrangement and beauty of design. We are glad that such a place of amusement has been opened to our citizens, where ladies and gentlemen can witness the pleasures of the trotting park, without encountering the rowdyism apt to be present at such places. We are certified that ample arrangements have been made to preserve the best of order and to absolutely exclude all persons of bad character from the grounds, making it a resort for ladies and gentlemen only. Therefore, success to the Driving Park and its managers.

THE ADVERTISER'S CITY EDITOR.—We discover now and then, in the periodical literature of the metropo lis literary productions emanating from the pen of Mr. Ausburn Towner. We are glad to note that Mr. Towner's talent as an easy, graceful writer is recognized and acknowledged abroad as well as at home. The descriptive letter press in the number of the *Daily Graphic*, in which Elmira was illustrated, was written by him, at the request of the editors of that journal, as was also a very entertainingly written sketch of Eldridge Park, for the *Christian Weekly*. Mr. Towner is also a frequent eontributor to other periodicals, in all of which he exhibits an originality of style that is very attractive and pleasing.

THE VINE STUB.—Our friends who have visited camp "Don't-Care-a-Darn," from year to year, will be sorry to learn that the beautiful old stub of a tree, covered with that magnificent woodbine, and which stood out in bold relief against the eastern sky, immediately across the creek from the camp, has fallen. Squire Bodine called to-day to convey the sorrowful tidings. For years, that old tree, with its graceful, green, drapery has stood, to be visited by hundreds who have laid down rod and flies to admire its comely proportions. Many are the artists, too, who carry its semblance in their sketch books, and who will search in vain for it next year; but to none will its loss be so much felt as to the campers, who used to sit about the camp-fire, smoked, chatted and commented upon the beauty of the stub as revealed against the sky in the soft twilight of evening.

CLARA MORRIS AND THE MOXA.—We have read much recently in the newspapers, of the terrrible suffering to which Clara Morris, the actress, was subjected by reason of the application of hot irons to her spine, (erroneously called the moxa), used for the purpose of arresting a spinal disease. The novelty and severeness of the operation has been extensively commented upon, as though the treatment of spinal irritation by hot irons was something entirely new and novel. The distinguished actress could have had the same treatment applied at home, without the necessity of seeking aid in a foreign land, had she called in any of the celebrated surgeons to be found in her own city. People make a great mistake in supposing that the best surgical talent is to be found in Paris and London, when the United States has become celebrated for her wonderful talent in surgery, and is acknowledged to be far in advance of all other countries in the appliances and skill of her surgeons. Prof. Gross' work on Surgery has been translated into nearly every language of the old world, and is everywhere recognized not only as a most excellent text book, but invaluable as a work of reference. New York city—the home of Miss Morris, has a score of surgeons any one of whom are the peers of the men who performed the very old operation of the actual cautery. Had she sought relief at home too, our own surgeons would have applied the *electro-cautery*, an American invention, by means of which the patient would have been spared much unnecessary suffering, for it is a well known fact to surgeons that a platinum point, heated to a *white* heat, by electricity, produces no pain in burning the flesh.

---

WROUGHT IRON FURNACES.—The season having arrived when it becomes necessary to provide heating apparatus for the winter, we heartily recommend the wrought iron furnace, as being the least objectionable of all the means for securing comfort by means of hot air. The wrought iron furnace, prevents the escape of gas and dust into the hot air chamber and secures an air free from the products of combustion. In cast iron furnaces, when hot, several deleterious gases escape through the red hot iron into the hot air chamber, to mix with the heated air that comes to our living apartments. These gases—carbonic acid, carbonic oxide, sulphurous acid gas—are highly deleterious, and produce the headaches and drowsiness to which people are subjected who are forced to live in such an atmosphere. The wrought iron furnace does away with all these objections, as the joints are riveted tightly and no gases can escape through this sort of iron. We are having a large sized furnace of this character introduced into the Institute and expect to give a good report of it in our next issue.

---

READING IN BED.—This is a proceedure that cannot be recommended for everyone, but to those who will adopt it under certain precautions, it is not only healthful, but extremely luxurious. By reason of exceedingly arduous duties during the day, we have adopted the plan of going to bed at 8 o'clock in the evening, more for rest than for sleep. We have an argand gas burner with blue chimney and shade, just back of our head, so that the light falls over our shoulder on the book as we lay bolstered up in bed. In this manner, we read or write until our eyes become weary or sleepy, when we turn out the gas and roll over for a refreshing sleep. One adopting this position for reading, will find it an improvement upon that of sitting upon the end of the spine, and shifting the body from time to time in order to secure any degree of comfort. Reclining in bed, rests the spine, soothes the nervous system, and prevents the exhausted feeling with which one seeks his bed after having passed the evening reading, while seated in a chair. Go to bed early, ye brain workers, who are forced to do reading at night; you will find the recumbent position will rest you, while the blue light will be agreeable to the eye.

---

SECTARIAN SCHOOLS.—Quite a furore has been created by reason of hiring the High Street School building, by the Board of Education, for use as an auxilliary school to District number one. This building was formerly used by our Catholic fellow citizens for a school exclusively for those of their own religious faith, but when leased by the board of education, was in the strongest terms possible, made entirely subservient to their rules and regulations, in which all exercises of a religious character were strictly prohibited, to the end that no "sectarianism" should creep into our public schools. It is claimed however, that the school is a Roman Catholic one, because the teachers happen to be of that faith, and because their religious observances prevent them accepting pay for their services, and that the sum appropriated for that purpose, is given to the priest of the parish, for the support of the church. Now, we *know* there is not a gentleman upon our Board of Education who would vote for sustaining a Catholic, or other sectarian school in our midst, and had no thought of so doing when they rented the premises referred to; neither did they enter into any agreement to appoint Catholics, Methodists or devotees of any other religious faith as teachers, nor did they stop to enquire what disposition was to be made of the money paid such individuals for honest labor performed, thinking if at all, that it was none of their business. Briefly, the Board of Education were pressed for room to accommodate the pupils in district number 1. The building known as the High St. School, was offered them at a fair rental, and being eligibly located, and commodious, it was accepted, and a school opened therein. Three "Sisters of St. Mary," a Catholic order, were appointed, not because they were Catholics, but because they were already teaching in the building, and were found to be unusually competent. They are strictly bound, by the terms of the lease not to offer any religious instruction, but to observe, in every particular, the rules and regulations of the Board of

Education. And this is all there is of the matter. If, upon examination, it is found they are violating the terms of the lease and *are* teaching their peculiar religious views, the school ought to be, aud no doubt *will* be, squelshed.

---

A PUPPY.—We have one. He's a thoroughbred, and knows a thing or two. He has a fine action, a good nose and irrepressible tongue. He's technically called a "setter," but *why*, is beyond finding out, for a more restless being could not well be found, and I doubt whether he was ever found "setting" for two consecutive minutes. The other day, he was found swinging on the clothes reel, in the back yard, having fastened his teeth in a finely embroidered garment of some sort, which he succeeded in tearing into shreds, while he grunted, growled and barked in very ecstacy of delight, until Laura—the maid, interrupted his hilarity with the broomstick. That piece of embroidery, consisting of a pair of short arms and a yoke, cost us, we were vexatiously informed, the sum of twelve dollars, and to have seen the manner in which that pup swung himself back and forth, and the alacrity with which he took a new hold every time a ribbon of it came away in his mouth, induced us to believe that it was worth the money. Yesterday, he eat, or in some other mysterious manner caused to disappear, the baby's best dress,—only a small portion of the lace which entered into its composition having yet been discovered, and was next observed in attempting a luncheon off of our patent leather boots, but only succeeded in tearing the legs from them, when further demolition was suspended, and that "likely pup" sent with a ki yi to his kennel. To-day, affairs culminated or were brought to a focus, so to speak, for when we entered our usually happy domicil, we discovered that a storm was brewing—in fact we thought we discovered a change in the atmosphere when a square away, but when our better-half met us at the dinner table, with fire in her eye and a determined expression of countenance, accompanied with these words: "that dog is a perfect abomination, and he must be chained up or taken away," it was easy to be perceived that "she meant business," so we sent for the necessary chain and collar before we dared to enquire what was up. It gradually leaked out that Zip had been viewing himself in the parlor mirror and while engaged in that commendable operation, had unconsciously knocked over and broken two elegant vases and carried their fragments to the yard, and when found, was industriously engaged in burying them among the choicest flowers, and so clumsy was he in the operation, that several geraniums, heliotropes, &c., were dug up and sacrificed in the operation. He then deliberately walked into the kitchen, with his muddy feet, and, when Laura's back was turned, jumped into the basket of clean clothes and snuggled down for a quiet snooze. Of course, he was dislodged—why certainly,—and in such a manner, that he carried a light of glass with him in his arduous attempt to comply with the wishes of the household to vacate speedily. Well, he's chained now, but this is far from being satisfactory, for he howls, barks, and raises the very d——l generally. Thrice daily, when at our meals, do we visit his kennel and apply the necessary chastisement, in order to teach him quietness. In the night time do we throw our boots, slippers and big cane at him, from our window, until he is almost persuaded that howling is not just the thing; for we observe that when he now gives a grunt, he looks aloft to see what manner of thing is coming from that window, and speculates upon the possibility of dodging it. But he is more afraid of the crack of a dog-whip than he would be to have a cook-stove hurled at him, and of this circumstance have we taken advantage and entered into a regular plot to deceive that knowing pup. We have procured a package of torpedoes, and when we retire for the night, we open the window and lay the pyrotechnics close at hand. When Zip barks,—bang, goes a torpedo near his head, and into the kennel he goes like a streak. When he thinks the coast clear, he stalks out cautiously, peers into the moonlight, and then ventures upon a mild sort of a bark, but with head pointed toward the kennel door. We throw another torpedo, with like result; and now, poor Zip, when he so far forgets himself as to offer a bark, waits for no invitation but immediately dives for that kennel and leaves us master of the situation. We admire that setter, the female portion of the household abhor him, the children love him, and the neighbors, well—they d——n him. And so the matter rests at present. But when we want another pup, be assured, he will be at least two years old and well broken.

---

SEARCH IN THE DARK.—Ever search for a strange lady, of a dark, rainy night, in a railway train, three miles from the station, (where the train is detained by an obstruction on the track), driving a skittish mare, over a new, uneven, and unknown road, and, after wading through the tall, wet weeds to the train, not find your expected individual aboard?—Ever try it? No?—Pshaw! Putting up a stove pipe's no circumstance to it, for delightful pastime.

